# Influenza and Pertussis Maternal Vaccination Coverage and Influencing Factors in Spain: A Study Based on Primary Care Records Registry

**DOI:** 10.3390/ijerph19074391

**Published:** 2022-04-06

**Authors:** María Isabel Fernández-Cano, Antonia Arreciado Marañón, Azahara Reyes-Lacalle, Maria Feijoo-Cid, Josep Maria Manresa-Domínguez, Laura Montero-Pons, Rosa Maria Cabedo-Ferreiro, Pere Toran-Monserrat, Gemma Falguera-Puig

**Affiliations:** 1 Department of Nursing, Faculty of Medicine, Universitat Autònoma de Barcelona, 08193 Bellaterra, Spain; mariaisabel.fernandezc@uab.cat (M.I.F.-C.); maria.feijoo@uab.cat (M.F.-C.); jmanresa@idiapjgol.info (J.M.M.-D.); 2 Grup de Recerca Multidisciplinar en Salut i Societat (GREMSAS), (2017 SGR 917), 08007 Barcelona, Spain; 3 Atenció a la Salut Sexual i Reproductiva de Sabadell, Direcció d’Atenció Primària Metropolitana Nord, Institut Català de la Salut, 08203 Barcelona, Spain; areyesl.ics@gencat.cat; 4 Research Group Atenció a la Salut Sexual i Reproductiva (GRASSIR), Institut Universitari d’Investigació en Atenció Primària Jordi Gol (IDIAP Jordi Gol), 08007 Barcelona, Spain; lmontero.ics@gencat.cat (L.M.-P.); rcabedo.ics@gencat.cat (R.M.C.-F.); gfalguera.mn.ics@gencat.cat (G.F.-P.); 5 Research Support Unit Metropolitana Nord, Primary Care Research Institut Jordi Gol (IDIAPJGol), 08303 Barcelona, Spain; ptoran.bnm.ics@gencat.cat; 6 Atenció a la Salut Sexual i Reproductiva de Santa Coloma de Gramenet, Direcció d’Atenció Primària Metropolitana Nord, Institut Català de la Salut, 08921 Barcelona, Spain; 7 Atenció a la Salut Sexual i Reproductiva de Granollers, Direcció d’Atenció Primària Metropolitana Nord, Institut Català de la Salut, 08400 Barcelona, Spain; 8 Department of Medicine, Faculty of Medicine, Universitat de Girona, 17071 Girona, Spain; 9 Atenció a la Salut Sexual i Reproductiva Metropolitana Nord, Direcció d’Atenció Primària Metropolitana Nord, Institut Català de la Salut, 08911 Barcelona, Spain

**Keywords:** antenatal care, influenza vaccine, maternally-acquired immunity, midwife, quantitative research, pertussis vaccine, pregnant vaccination

## Abstract

The purpose was to determine the coverage of maternal vaccination against influenza and pertussis, and the characteristics associated with being vaccinated, in a health area of Catalonia, Spain. Some 36,032 anonymized and computerized clinical records registries of pregnant women from Primary Care Centres (e-CAP database) were analysed, from between 2015 and 2018. Vaccination coverage and the association with sociodemographic variables and clinical conditions were estimated using a Poisson regression model. Maternal vaccination coverage against influenza ranged between 11.9% in 2015 and 6.8% in 2018, following a decreasing trend (*p* < 0.001). Coverage with the tetanus toxoid, diphtheria toxoid, and acellular pertussis vaccine varied between 49.8% in 2016 and 79.4% in 2018, following an increasing trend (*p* < 0.001). Having living children and suffering from obesity were factors associated with not being vaccinated against both infections. The predictive variables of vaccination against influenza were diabetes (IRR: 2.17, 95% CI: 1.42–3.30) and asthma (IRR: 2.05, 95% CI: 1.76–2.38); and for pertussis, it was asthma (IRR: 1.10, 95% CI: 1.03–1.17). Different socio-demographic factors and chronic conditions in pregnant women were associated with maternal vaccination, and which will have to be taken into account in clinical practice when implementing strategies to improve the coverage of the programme.

## 1. Introduction

Respiratory infections due to influenza and pertussis cause significant morbidity and mortality in pregnant women and neonates. The WHO recommends maternal vaccination (MV) against influenza and pertussis to prevent the consequences arising from mothers and neonates being infected [1,2]. It is estimated that influenza infection seriously affects three to five million people annually, causing between 290,000 and 650,000 deaths [3]. Pregnant women have the same risk of suffering from influenza as other women, but they have a higher risk of presenting a severe clinical course with complications. The risk of a pregnant woman being hospitalized with influenza is 2.4 times higher than the rest of the population [4]. In the US, between 2010 and 2018, pregnant women accounted for 24% to 34% of hospitalizations associated with seasonal influenza among women of childbearing age. In Spain, in the same period, it was seen that pregnant women hospitalized for influenza had an increased risk of admission to the ICU and death by 2.43 times more compared to non-pregnant women [5]. In addition, the infection can lead to complications such as premature delivery, low birth weight, and death of the newborn [6,7].

The WHO estimated that in 2018 there were just over 151,000 cases of pertussis infection [8], despite the high immunization coverage of the population with three doses of tetanus toxoid, diphtheria toxoid, and acellular pertussis (DTaP) vaccine (86%). Children under one year of age have the highest risk of serious complications (50% of cases are hospitalized) and death (90% of deaths that occur) due to this cause [9], with their main source of contagion being the cohabitants of their environment, especially the mother [10].

MV programmes protect mothers and infants passively and actively (due to the passage of antibodies through the placenta) [11]. MV against influenza is safe and effective, since it reduces the number of cases in pregnant women and the severity of influenza in those cases in which it does not prevent infection [5]. At the same time, it is 71% effective in preventing infections and 64% effective in preventing hospitalizations due to influenza in the neonates of vaccinated pregnant women [12]. MV against pertussis has also been shown to be safe [13] and highly effective (91%) in preventing cases in neonates [14]. In addition, evaluations of the MV strategy show it to be cost-effective, both for influenza [15] and pertussis [16]. 

However, not all countries recommend MV. The Advisory Committee on Immunization Practices (ACIP) has recommended MV against seasonal influenza since 2004 [17] and the tetanus toxoid, diphtheria toxoid, and acellular pertussis (Tdap) vaccine in each pregnancy since 2012 [18]. MV coverage against influenza and pertussis in the US has remained slightly above 50% [19]. In Europe, vaccination against influenza for risk groups and health personnel has been recommended since 2009 [20] and since 2012, MV has been incorporated into the vaccination plans of most countries (28 countries) [21]. However, vaccination coverage for risk groups is far from the target set (75%) [20], with a mean coverage of 25% in pregnant women [21]. MV against pertussis is recommended in eight European countries [22]. In the United Kingdom, vaccination coverage against pertussis reached 74.2% in 2017 [23]. 

In Spain, MV against influenza has been recommended since 2005, but coverage is low. According to data from the Ministry of Health, for the 2018–19 campaign a coverage of 50% was estimated with large differences between regions (from 22.7% to 65%) [24]. In the same year, MV coverage with Tdap was 83% with variations between regions (from 56.6% to 95.1%) [25]. Despite the evidence that MV against influenza and pertussis is safe and effective, and recommendations by health professionals and authorities across the country, coverage has remained below desirable figures. In Spain, studies on the characteristics of pregnant women who are not vaccinated are scarce and it would be interesting to be able to compare them with the published results. Knowing the characteristics of pregnant women who are not vaccinated will guide the improvement of vaccination coverage strategies.

The objective of the study was to determine the coverage of MV against influenza and pertussis, as well as the characteristics associated with being vaccinated, in a health area of Catalonia, Spain.

## 2. Materials and Methods

The anonymized and computerized records of pregnant women in the North Metropolitan Health Area of the province of Barcelona (Catalonia), Spain were analysed. The area serves 1,400,000 citizens from 70 municipalities (rural and urban) [26] with an immigrant population of 15% [27]. All citizens in Catalonia are assigned a Primary Care Centre (CAP) and a Sexual and Reproductive Health Care Centre (ASSIR) where they are cared for by teams of health professionals. The records of women with a confirmed pregnancy diagnosis (ICD Z32.1), who visited the seven ASSIR centres or the CAPs in the period from 2 January 2015 to 31 December 2018, were included.

In Spain, vaccines against influenza and pertussis are publicly financed and dispensed in the CAPs and ASSIRs. It is estimated that 20% of pregnant women are not attending ASSIR centres with their pregnancies either because they are high-risk pregnancies that require care in specialized hospitals, because they choose private centres, or because there is no kind of follow-up [28]. The monitoring of healthy pregnant women is carried out in the ASSIR centres by a midwife, a graduate nurse with a two-year specialization, following the recommendations of the pregnancy monitoring protocol in Catalonia, which includes MV against influenza and pertussis. Pregnant women are informed of vaccination against influenza and pertussis, both in maternal education sessions and in consultations. Administration of the Tdap vaccine is recommended between weeks 27 and 36 of gestation. The influenza vaccination campaign is carried out annually between the beginning or mid-October and the end of January, and vaccination is recommended for pregnant women during this period, regardless of their stage of pregnancy. The midwives themselves or the CAP nurses are responsible for administering the vaccine and completing the records, depending on the organization of each centre, without the need for a prescription from a medical professional. The government of Catalonia established a financial incentive for midwives from 2017 onwards in order to help reach the MV coverage target established in the health plan.

A descriptive analysis of the socio-demographic variables was carried out: age, mother’s place of birth, father’s place of birth, parents’ educational level (taking the highest educational level of the parents as the value); and of the clinical variables: obstetric history, chronic diseases in the mother that increased the risk of complications in case of infection by influenza or whooping cough (morbid obesity ICD: E66.8), type 1 and 2 diabetes (ICD: E10.9 and E11.9), hypertension (ICD: I10), asthma (ICD: J45), COPD (ICD: J44.9) and smoking (CIE: F17.200), MV against influenza and MV against pertussis. Global coverage of MV against influenza and pertussis was estimated according to socio-demographic variables. For pregnant women with monitoring in the ASSIR centres, vaccination coverage against influenza was estimated using as a denominator the number of pregnant women who had any moment of their gestation coinciding with the seasonal influenza vaccination campaign (excluding those pregnant women whose date of the last period was between 1 and 20 January). 

For each vaccine, the incidence rate ratios (IRR) and its 95% CI of having been vaccinated or not during pregnancy (outcome) with respect to socio-demographic and clinical variables were studied using Poisson regression models. In the first bivariate regression models, we identified variables that were individually associated with the administration of each vaccine at *p* ≤ 0.20. These variables were then included in multivariate models. The variables that had more than 50% of data missing (autochthonous parents, educational level, and habit of smoking) were not included.

The level of significance was 5%. The analyses were performed with the Stata statistical package for Windows, version 16.0.

The Clinical Research Ethics Committee of the Primary Care Research Institute Jordi Gol assessed and approved the project (P18/007).

## 3. Results

A total of 36,032 records of pregnant women were included in the analysis, of which 32,606 records (90.5%) came from pregnant women with monitoring information in Sexual and Reproductive Health Care centres and 3426 records (9.5%) with evidence of an active pregnancy but without monitoring in Sexual and Reproductive Health Care centres. The mean age of the pregnant women was 31.6 years (SD 5.6) and 5600 (15.6%) of them were of foreign origin. Table 1 shows the socio-demographic and clinical variables of the pregnant women according to the registry of maternal vaccination against influenza and pertussis. Some 3356 pregnant women (9.31%) were vaccinated against influenza, 22,918 pregnant women (63.6%) with Tdap vaccine, and 1917 pregnant women (5.3%) were vaccinated against both. The coverage of maternal vaccination against influenza in pregnant women with monitoring in Sexual and Reproductive Health Care centres whose gestation coincided with the annual vaccination campaign was 6.4% (1952 of 30,709 pregnant women).

**Figure 1:**
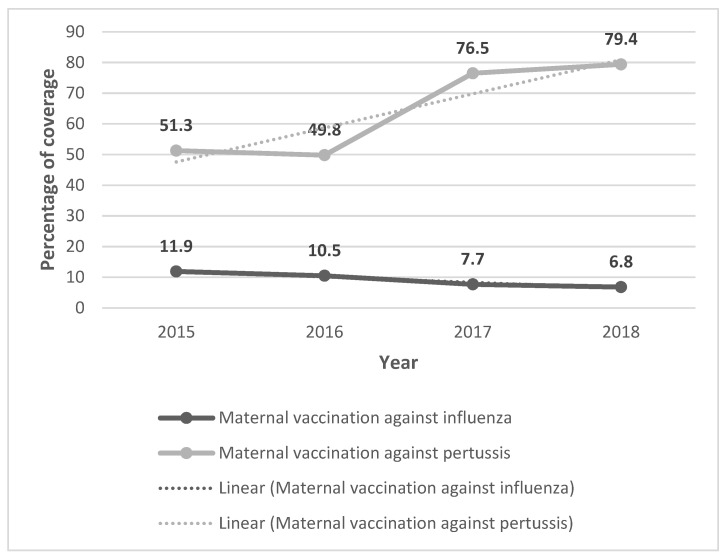
Percentage of maternal vaccination coverage against influenza and pertussis and the linear trend according to year.

The global coverage of maternal vaccination against influenza ranged from 11.9% in 2015 to 6.8% in 2018, following a statistically significant decreasing linear trend (IRR = 0.82, *p* < 0.001) (Figure 1). Maternal vaccination coverage with Tdap ranged from 49.8% in 2016 to 79.4% in 2018, following a statistically significant increasing trend (IRR = 1.19; *p* < 0.001). Some 99.2% of maternal vaccinations for pertussis were performed in the correct weeks of gestation.

There were 6051 pregnant women with risk conditions (16.8%), with 595 (10%) presenting more than one pregnancy risk condition. Obesity and smoking were the most frequent risk conditions, 12.5%, and 5.2% respectively. The highest proportion of pregnant women who were not vaccinated and who had risk conditions were those with diabetes, at 56% (98/175). The frequencies and percentages of vaccination against influenza and pertussis according to the presence of risk conditions are shown in Table 2.

The variables associated with MV against influenza were the age of more than 25 years old, autochthonous mother and father, a university level of education, and suffering from asthma. Pregnant women with living children and obese women are respectively 22% and 30% less likely to be vaccinated against influenza than those without these conditions (Table 3). The variable most associated with MV against pertussis was asthma. Pregnant women with live children and those with morbid obesity, diabetes, or hypertension are less likely to be vaccinated against pertussis than those without these conditions. Pregnant women with diabetes and pregnant women with hypertension have 39% and 18% less likelihood of vaccination against pertussis, respectively (Table 3).

In the multivariate analysis with the Poisson model, a slight decrease in the coverage of MV against influenza is observed over the years, while the coverage of MV against pertussis increases progressively from 2017, with it being 50% higher in 2018 than in 2015.

The predictive variables of MV against influenza were diabetes (IRR: 2.10, 95% CI: 1.37–3.21) or asthma (IRR: 2.03, 95% CI: 1.74–2.36), and for MV against pertussis it was only asthma (IRR: 1.10, 95% CI: 1.03–1.17) However, diabetes (IRR: 0.66, 95% CI: 0.52–0.84) or hypertension IRR: 0.86, 95% CI: 0.76–0.98 were predictive variables of no maternal vaccination against pertussis. Having living children was a predictor of no maternal vaccination for both vaccines (Table 4).

## 4. Discussion

In the study, MV coverage against influenza and pertussis was estimated, and socio-demographic and clinical variables were analysed as predictors of vaccination.

### 4.1. Maternal Vaccination Coverage against Influenza

MV coverage against influenza was insufficient (between 6.8% and 11.9%), well below the target set (75%) [20]; This is in stark contrast to the US [19] and European [21] results, where MV coverage against influenza averages are 53.7% and 25%, respectively, exceeding 50% in the United Kingdom and Ireland. The effect of the COVID-19 pandemic may have positively influenced the propensity to be vaccinated against seasonal flu [29]. In Catalonia during 2020–21, the percentage of vaccinated pregnant women rose to 41.7% (61.9% in Spain)—showing a significant improvement—although it continues to be below the established target [30]. In the US, an increased rate of vaccination from 2019 to 2020 was also reported, rising to 61.2%. However, in England, a large reduction in vaccination rates of up to 43.7% was recorded [31]. The variability of acceptance of the vaccine against COVID-19 in pregnant women is also wide, between 29.7% and 77.4%, geographically dependent, and related to acceptance of the influenza vaccine [32]. These results suggest that promoting MV against COVID-19 together with influenza may be beneficial for increasing vaccination coverage of the latter. 

The strategies that obtained the best results for increasing MV coverages in developed countries were to train patients and health professionals, and allow midwives to administer vaccines without prior prescription from a medical professional [23]. In Spain, unlike other countries, both midwives and nursing professionals administer vaccines without the need for a medical professional, but coverage is not as high as might be expected, above all in the case of influenza. Among healthcare professionals, vaccination coverage against influenza is low [33], especially in the group of nurses [34] and midwives [35], and this may mean recommendation was not made with the same intensity. Furthermore, in our context, achieving higher coverage in the case of MV against influenza has not been set as an incentivized target for health professionals. There are numerous publications that show the positive effect of having a professional recommend and administer vaccines. In Australia, the intervention of midwives in the MV programme against influenza increased the probability that pregnant women were vaccinated by eight times [36]. In the US, coverage for influenza vaccination for pregnant women who had vaccination recommended to them and who were offered it reached 65.7% [19]. 

Among the reasons reported by pregnant women for not getting vaccinated against influenza would be the belief that the vaccine is not effective and not safe for the foetus [19]. Some socio-demographic adjustment variables were predictors of MV. The age of the mother, over 25 years old, turned out to be the most determining factor for MV against influenza. Similar results were obtained in a study carried out in Australia [37]. In the case of associating parental origin and level of studies with MV, results must be evaluated with caution, given the high percentage of unknown data that was recorded. The foreign origin of the pregnant women and their partners determined a lower MV for the influenza vaccine. Laenen et al. obtained the same results, hypothesizing that low linguistic competence would make it difficult to understand the indications of health professionals [38]. However, cultural and ethnic factors can also explain this result. In a recent study [39], differences were found in the perception of the risk of acquiring the infection and confidence in the safety of vaccines during pregnancy according to the race and origin of the pregnant women. Thus, black and Hispanic pregnant women perceived less risk of becoming infected and showed less confidence in MV compared to white pregnant women. MV against influenza was positively associated with the educational level of parents, with university students having the highest coverage, perhaps due to better access to information about the vaccine and its advantages. 

A recent meta-analysis [40] found that having previous children significantly reduced MV against influenza. These results coincide with those obtained in previous studies, both in our country [41] and in other contexts [36]. It seems that there are differences between new mothers and mothers with previous children with regards to their experience of pregnancy and in their concern for the health of their future child [42]. Women who have given birth previously would worry less overall, and that could be one of the reasons they get vaccinated less. 

In Spain, 8.8% of the women who were having their first child were 40 years of age or older [43], which may mean that a higher proportion of them have chronic underlying diseases. It is striking that pregnant women with chronic diseases, who would have additional reasons for vaccination against influenza, show significantly lower coverage. These results coincide with others published [44], although it is not clear what the reasons for not being vaccinated could be. A possible explanation, in the case of morbidly obese pregnant women, could be the relationship between obesity and a lower level of education [45]. This association would be consistent with the results obtained, which show a positive association between vaccination against influenza and a higher level of studies. However, they contrast with those obtained in a meta-analysis [40] in which pregnant women with risk conditions were vaccinated 30% more than those without. Similar results were obtained with respect to suffering from asthma. 

### 4.2. Maternal Vaccination Coverage against Pertussis

MV coverage against pertussis was low (between 49.8% and 79.4%) but with a tendency towards increasing. Again, this is in stark contrast to the results from the USA [19] where MV coverage against pertussis is 54.9% and other European countries—such as the United Kingdom and Belgium [46]—where coverage of MV against pertussis reaches values higher than 60%. In 2020, the year of the COVID-19 pandemic, Catalonia registered a higher vaccination coverage against pertussis of 86.9% (85.4% in Spain) [30]. However, in England, in 2021 the mean coverage was 64.9%, 1.8% lower than in 2020 [47]. The positive effect of having a professional recommend and administer vaccines was the same as in the case of influenza MV. In Australia, the intervention of midwives in the MV programme against pertussis increased the probability of pregnant women being vaccinated by 32 times [36]. In the US, coverage for pertussis vaccination for pregnant women who had vaccination recommended to them and who were offered it reached 70.5% [19]. Pregnant women point out recommendation by the healthcare professional of reference [35] as the most decisive factor in being vaccinated; even those pregnant women who did not get vaccinated admitted that they would have done so if it had been recommended [48]. The greater recommendation for MV against pertussis by midwives in our study may be due to the fact that this was set as a financially incentivized target from 2017 onwards, in order to achieve coverage of greater than 50% (internal unpublished document). The main reason stated by pregnant women for not being vaccinated against pertussis was not knowing that they needed to be vaccinated for each pregnancy [19]. The age of the mother and the educational level were not a predictive factor in the case of vaccination against pertussis. These results are similar to those of other studies [38]. Educational level is not a definitive indicator of health literacy, but it is higher in people with a higher educational level [49]. Maternal health literacy is a determining factor in compliance with the recommendations and in the health outcomes of pregnant women and children [50]. Midwives are identified by pregnant women as the professionals who offer the most recommendation for MV [48] and should have the necessary resources to adapt information to patients based on their level of education. As in the case of influenza MV having previous children seems to be a predictive factor for no MV.

Most scientific publications highlight positive aspects of vaccination, both against influenza and pertussis [51]. Also, the main sources of information on social networks in Spanish show a positive tone towards MV [52]. However, publications on pertussis vaccination have an impact on the protection of the newborn while publications on influenza vaccination do so on the protection of the mother. Given this positive attitude towards MV, professionals should highlight the protective effects for both the mother and the newborn of both vaccines.

As possible limitations of this study, it should be noted that all available records, real data, have been included in the analysis, although the information was incomplete for three socio-demographic adjustment variables, with proportions of unknown data of 63.5% for the origin of the mother, 74.7% for the origin of the father and 87.3% for the educational level of the parents. Completion of healthcare records is low. The evidence indicates that medical records have a high prevalence of unknown data; as such, in cancer registry data, it was seen that variables of clinical interest had between 39.7% and 71% unknown data [53]. Some nursing professionals perceive the implementation of registries as a task of little use that takes time away from patient care [54]. There are few studies in our context that allow for an estimation of the weight of the factors that condition MV and therefore it was decided to include all records. In addition, the analysis included pregnant women who carried out pregnancy monitoring at ASSIR centres or who went to the Primary Care Centre to get vaccinated. Those that were monitored in private centres, in specialized hospital centres because they were high-risk pregnancies, or vulnerable groups that did not attend ASSIR centres were probably excluded. The fact that vaccines are easily accessible and free within the National Health System means that vaccinations outside the public system are very rare, so it is assumed that the estimates have a very small margin of error.

Improving the quality of records in computerized medical records is essential to advance research, improve clinical practice, and increase knowledge. Improving MV coverage would imply that health professionals, especially midwives as those mainly responsible for monitoring pregnant women, receive more training on the safety and benefits of vaccines during pregnancy, especially the influenza vaccine. In addition, establishing a financially incentivized target for midwives to increase MV, both against whooping cough and influenza, would help improve coverage. Based on the results, more attention should be paid in clinical practice to foreign pregnant women, pregnant women with a low educational level, and pregnant women with previous chronic diseases.

## 5. Conclusions

This study offers an estimate of recent MV coverage against influenza and pertussis in Spain. The results of the study may be helpful in guiding strategies for improving MV at the level of health professionals, institutions, and public health policies.

MV coverages are lower than recommended and while vaccination coverage against pertussis has tended to improve (especially from 2017 onwards), coverage against influenza is decreasing. Various socio-demographic factors and clinical situations of the pregnant women were associated with MV. In healthcare practice, the priority would be to increase the recommendation of vaccines by midwives to pregnant women with these characteristics. Updating the knowledge of these professionals should be promoted, with regards to scientific evidence on the effectiveness and safety of vaccines, and awareness of the serious forms that influenza and pertussis infections can present, both in pregnant women and in newborns.

Policies to improve MV coverage should include setting financially incentivized targets for midwives to recommend more MV, especially against influenza.

## Figures and Tables

**Table 1:** Demographic and clinical characteristics of pregnant women vaccinated against influenza and pertussis, from 2015 to 2018 in a health area of Catalonia, Spain.

Demographic and Clinical Variables	Total *n* = 36,032 Frequency (%)	Vaccinated against Influenza *n* = 3356 Frequency (%)	Vaccinated against Pertussis *n* = 22,918 Frequency (%)
Year			
2015	9295 (25.8)	1103 (32.9)	4769 (20.8)
2016	9563 (26.5)	1006 (30.0)	4762 (20.8)
2017	8726 (24.2)	672 (20.0)	6676 (29.1)
2018	8448 (23.4)	575 (17.1)	6711 (29.3)
Age (average, SD)	31.6 (5.6)	33.7 (5.2)	31.5 (5.6)
Age Group			
<18	246 (0.7)	12 (0.4)	146 (0.6)
18–24	4153 (11.5)	218 (6.5)	2647 (11.5)
25–37	26,460 (73.4)	2525 (75.2)	16,849 (73.5)
>37	5171 (14.4)	601 (17.9)	3276 (14.4)
Unknown	2 (0.0)		
Place of birth of the mother			
Spain	7539 (20.9)	490 (14.6)	5804 (25.3)
Abroad	5600 (15.6)	287 (8.6)	4169 (18.2)
Unknown	22,893 (63.5)	2579 (76.8)	12,945 (56.5)
Place of birth of the father			
Spain	5267 (14.6)	391 (11.7)	4203 (18.3)
Abroad	3840 (10.7)	213 (6.3)	2976 (13.0)
Unknown	26,925 (74.7)	2752 (82.0)	15,739 (68.7)
Level of education of the parents			
Primary	1358 (3.8)	85 (2.5)	1068 (4.7)
Secondary	1212 (3.4)	87 (2.6)	1005 (4.4)
Professional training	1077 (3.0)	86 (2.6)	869 (3.8)
University	921 (2.6)	85 (2.5)	718 (3.1)
Unknown	31,464 (87.3)	3013 (89.8)	19,258 (84.0)
Obstetric history ^1^			
Full-term pregnancies	21,973 (61.0)	1212 (62.2)	13,698 (66.6)
Premature births	1223 (3.4)	74 (3.8)	703 (3.4)
Abortions	11,632 (32.3)	662 (34.0)	7238 (35.2)
Live children	22,397 (62.2)	1233 (63.3)	13,954 (67.8)
Risk conditions			
No	29,981 (83.2)	2882 (85.9)	19,134 (83.5)
Yes	6051 (16.8)	474 (14.1)	3784 (16.5)

^1^ Each pregnant woman can be included in more than one option.

**Table 2:** Maternal vaccination against influenza and whooping cough according to maternal risk conditions.

Risk Conditions	Frequency (%)	Not Vaccinated *N* (%)	Vaccinated against Influenza *N* (%)	Vaccinated against Pertussis *N* (%)	Vaccinated against Influenza and Pertussis *N* (%)
Morbid obesity	4486 (12.5)	1679 (37.4)	62 (1.4)	2502 (55.8)	243 (5.4)
Chronic Obstructive Pulmonary Disease	24 (0.1)	11 (45.8)	0 (0)	12 (50.0)	1 (4.2)
Asthma	1570 (4.4)	445 (28.3)	42 (2.7)	942 (60.0)	141 (9.0)
Diabetes (type 1 and 2)	175 (0.5)	98 (56.0)	9 (5.1)	55 (31.4)	13 (7.4)
Hypertension	436 (1.2)	199 (45.6)	8 (1.8)	205 (47.0)	24 (5.5)
Smoker	1867 (5.2)	359 (19.2)	7 (0.4)	1394 (74.7)	107 (5.7)

**Table 3:** Bivariate Poisson Regression analysis to determine the predictive variables of maternal vaccination against influenza and pertussis.

			Vaccination against Influenza		Vaccination against Pertussis	
	*N*	*n* (%)	IRR ^1^ (95% CI)	*p*	IRR ^1^ (95% CI)	*p*
Year	36,032					
2015		9295 (25.8)	Reference		Reference	
2016		9563 (26.5)	**0.89 (0.81–0.97)**	**0.006**	0.97 (0.93–1.01)	0.145
2017		8726 (24.2)	**0.65 (0.59–0.71)**	**0.000**	**1.07 (1.44–1.55)**	**0.000**
2018		8448 (23.4)	**0.57 (0.52–0.63)**	**0.000**	**1.55 (1.49–1.61)**	**0.000**
Age	36,030					
<18		246 (0.7)	Reference		Reference	
18–24		4153 (11.5)	1.08 (0.60–1.92)	0.805	1.07 (0.91–1.27)	0.401
25–37		26,460 (73.4)	**1.96 (1.11–3.45)**	**0.020**	1.20 (0.91–1.26)	0.397
>37		5171 (14.4)	**2.38 (1.35–4.22)**	**0.003**	1.18 (0.90–1.26)	0.440
Autochthonous mother	13,139	7539 (57.4)	**1.27 (1.10–1.47)**	**0.001**	1.03 (0.99–1.08)	0.098
Autochthonous father	9107	5267 (57.8)	**1.34 (1.13–1.58)**	**0.001**	1.03 (0.98–1.08)	0.222
Level of education of the parents	4568					
Primary		1358 (29.7)	Reference		Reference	
Secondary		1212 (26.5)	1.15 (0.85–1.55)	0.369	1.05 (0.97–1.15)	0.228
Professional		1077 (23.6)	1.28 (0.95–1.72)	0.111	1.03 (0.94–1.12)	0.575
University		921 (20.2)	**1.47 (1.09–1.99)**	**0.011**	0.99 (0.90–1.10)	0.856
Live children	32,535	22,397 (68.8)	**0.78 (0.71–0.86)**	**0.000**	**0.95 (0.93–0.98)**	**0.001**
Obesity	36,032	4486 (12.5)	**0.70 (0.62–0.79)**	**0.000**	**0.96 (0.92–1.00)**	**0.030**
COPD	36,032	24 (0.1)	0.45 (0.06–3.18)	0.421	0.85 (0.49–1.47)	0.562
Diabetes	36,032	175 (0.5)	1.35 (0.89–2.06)	0.159	**0.61 (0.48–0.77)**	**0.000**
Hypertension	36,032	436 (1.2)	0.79 (0.55–1.11)	0.175	**0.82 (0.72–0.94)**	**0.004**
Asthma	36,032	1570 (4.4)	**1.27 (1.10–1.47)**	**0.002**	**1.09 (1.02–1.16)**	**0.006**
Smoker	11,740	1867 (15.9)	0.98 (0.80–1.19)	0.820	1.04 (0.98–1.10)	0.190

^1^ IRR increase of relative risk. Significant IRRs (95% CI) have been indicated in bold.

**Table 4:** Multivariate Poisson Regression models with the predictive variables of maternal vaccination against influenza and pertussis.

Final Models	Maternal Vaccination against Influenza		Maternal Vaccination against Pertussis	
	IRR ^1^ (95% CI)	*p*	IRR ^1^ (95% CI)	*p*
Year				
2015	Reference		Reference	
2016	0.85 (0.76–0.96)	**0.009**	0.97 (0.93–1.01)	0.134
2017	0.71 (0.62–0.81)	**<0.001**	1.49 (1.43–1.55)	**<0.001**
2018	0.92 (0.82–1.04)	0.208	1.53 (1.47–1.59)	**<0.001**
Age				
<18	Reference		Reference	
18–24	1.13 (0.59–2.15)	0.709	1.05 (0.89–1.25)	0.545
25–37	1.58 (0.84–2.96)	0.152	1.08 (0.91–1.27)	0.373
>37	1.82 (0.97–3.44)	0.064	1.08 (0.91–1.28)	0.401
Live children	0.75 (0.68–0.82)	**<0.001**	0.97 (0.94–1.00)	**0.029**
Obesity	1.13 (1.00–1.28)	0.059	1.01 (0.97–1.05)	0.708
COPD	0.51 (0.07–3.74)	0.510	1.02 (0.59–1.77)	0.937
Diabetes	2.10 (1.37–3.21)	**0.001**	0.66 (0.52–0.84)	**0.001**
Hypertension	1.05 (0.74–1.50)	0.778	0.86 (0.76–0.98)	**0.026**
Asthma	2.03 (1.74–2.36)	**<0.001**	1.10 (1.03–1.17)	**0.003**
Constant	0.05		0.49	

^1^ IRR increase of relative risk. Significant IRRs (95% CI) have been indicated in bold.

## Data Availability

Restrictions apply to the availability of these data. Data was obtained from Primary Care Research Institute IDIAP Jordi Gol and the use of data was only for this study.

## References

[1] World Health Organization (2019). Global Influenza Strategy 2019–2030. https://apps.who.int/iris/bitstream/handle/10665/311184/9789241515320-eng.pdf?ua=1.

[2] World Health Organization (WHO) (2016). Pertussis Vaccines: WHO Position Paper, August 2015-Recommendations. Vaccine.

[3] Iuliano A., Roguski K.M., Chang H.H., Muscatello D.J., Palekar R., Tempia S., Mustaquim D. (2018). Estimates of global seasonal influenza-associated respiratory mortality: A modelling study. Lancet.

[4] Mertz D., Geraci J., Winkup J., Gessner B.D., Ortiz J.R., Loeb M. (2017). Pregnancy as a risk factor for severe outcomes from influenza virus infection: A systematic review and meta-analysis of observational studies. Vaccine.

[5] Godoy P., Romero A., Soldevila N., Torner N., Jané M., Martínez A., Domínguez A. (2018). Influenza vaccine effectiveness in reducing severe outcomes over six influenza seasons, a case-case analysis, Spain, 2010/11 to 2015/16. Eurosurveillance.

[6] Pierce M., Kurinczuk J.J., Spark P., Brocklehurst P., Knight M., UK Obstetric Surveillance System (UKOSS) (2011). Perinatal outcomes after maternal 2009/H1N1 infection: National cohort study. BMJ.

[7] Håberg S.E., Trogstad L., Gunnes N., Håberg S.E., Trogstad L., Gunnes N., Wilcox A.J., Gjessing H.K., Samuelsen S.O., Stoltenberg C. (2013). Risk of fetal death after pandemic influenza virus infection or vaccination. N. Engl. J. Med..

[8] World Health Organization Health Topics. Pertussis. https://www.who.int/health-topics/pertussis#tab=tab_2.

[9] Skoff T.H., Hadler S., Hariri S. (2019). The epidemiology of nationally reported pertussis in the United States, 2000–2016. Clin. Infect Dis..

[10] Wiley K.E., Zuo Y., Macartney K.K., McIntyre P.B. (2013). Sources of pertussis infection in young infants: A review of key evidence informing targeting of the cocoon strategy. Vaccine.

[11] Vilajeliu A., Goncé A., López M., Costa J., Rocamora L., Ríos J., Teixidó I., Bayas J.M. (2015). Combined tetanus-diphtheria and pertussis vaccine during pregnancy: Transfer of maternal pertussis antibodies to the newborn. Vaccine.

[12] Dabrera G., Zhao H., Andrews N., Begum F., Green H.K., Ellis J., Elias K., Donati M., Zambon M., Pebody R. (2014). Effectiveness of seasonal influenza vaccination during pregnancy in preventing influenza infection in infants, England, 2013/14. Euro. Surveill..

[13] McMillan M., Clarke M., Parrella A., Fell D.B., Amirthalingam G., Marshall H.S. (2017). Safety of Tetanus, Diphtheria, and Pertussis Vaccination During Pregnancy: A Systematic Review. Obstet. Gynecol..

[14] Amirthalingam G., Andrews N., Campbell H., Ribeiro S., Kara E., Donegan K., Fry N., Miller E., Ramsay M.E. (2014). Effectiveness of maternal pertussis vaccination in England: An observational study. Lancet.

[15] Xu J., Zhou F., Reed C., Chaves S.S., Messonnier M., Kim I.K. (2016). Cost-effectiveness of seasonal inactivated influenza vaccination among pregnant women. Vaccine.

[16] Fernández-Cano M.I., Armadans L., Campins M. (2015). Cost-benefit of the introduction of new strategies for vaccination against pertussis in Spain: Cocooning and pregnant vaccination strategies. Vaccine.

[17] Harper S.A., Fukuda K., Uyeki T.M., Cox N.J., Bridges C.B. (2004). Centers for Disease Control and Prevention (CDC) Advisory Committee on Immunization Practices (ACIP). Prevention and control of influenza: Recommendations of the Advisory Committee on Immunization Practices (ACIP). MMWR Recomm. Rep. Morb. Mortal. Wkly. Rep. Recomm. Rep..

[18] Centers for Disease Control and Prevention Pregnancy and Vaccination. https://www.cdc.gov/vaccines/pregnancy/hcp-toolkit/important-maternal-vaccines.html.

[19] Lindley M.C., Kahn K.E., Bardenheier B.H., D’Angelo D.V., Dawood F.S., Fink R.V., Havers F., Skoff T.H. (2019). Vital Signs: Burden and Prevention of Influenza and Pertussis Among Pregnant Women and Infants—United States. MMWR Morb. Mortal. Wkly. Rep..

[20] Council of the European Union (2009). Council Recommendation of 22 December 2009 on seasonal influenza vaccination. Off. J. Eur. Union.

[21] Mereckiene J., European Centre for Disease Prevention and Control Seasonal Influenza Vaccination and Antiviral Use in EU/EEA Member States—Overview of Vaccine Recommendations for 2017–2018 and Vaccination Coverage Rates for 2015–2016 and 2016–2017 Influenza Seasons. https://www.ecdc.europa.eu/sites/default/files/documents/Seasonal-influenza-antiviral-use-EU-EEA-Member-States-December-2018_0.pdf.

[22] Mereckiene J., Cotter S., Nicoll A., Lopalco P., Noori T., Weber J.T., O’Flanagan D. (2014). Seasonal influenza immunisation in Europe. Overview of recommendations and vaccination coverage for three seasons: Pre-pandemic (2008/09), pandemic (2009/10) and post-pandemic (2010/11). Euro Surveill..

[23] Bisset K.A., Paterson P. (2018). Strategies for increasing uptake of vaccination in pregnancy in high-income countries: A systematic review. Vaccine.

[24] Ministry of Health, Consumption and Social Welfare—Government of Spain Evolution of Primary Vaccination Coverage. Spain 2008–2018. Vaccines and Vaccination Programs. Vaccination Coverage. https://www.sanidad.gob.es/profesionales/saludPublica/prevPromocion/vacunaciones/calendario-y-coberturas/coberturas/docs/Todas_las_tablas2018.pdf.

[25] Ministry of Health, Government of Spain Vaccination Coverage with dTpa in Pregnant Women. Autonomous Communities. 2020. Vaccines and Vaccination Programs. Vaccination Coverage. https://www.mscbs.gob.es/profesionales/saludPublica/prevPromocion/vacunaciones/calendario-y-coberturas/coberturas/docs/Tabla12.pdf.

[26] Government of Catalonia, Catalan Institute of Health Memòria Metropolitana Nord. http://ics.gencat.cat/ca/lics/memories-dactivitat/memories-territorials/metropolitana-nord/.

[27] Institute of Statistics of Catalonia (IDESCAT) Statistical Yearbook of Catalonia. Population. https://www.idescat.cat/tema/xifpo.

[28] Generalitat of Catalonia, Health Department Plan Estratégico de Ordenación de Servicios de La Atención a La Salud Sexual y Reproductiva. https://assirbarcelonaics.wordpress.com/2014/02/27/que-es-lassir/.

[29] Domnich A., Cambiaggi M., Vasco A., Maraniello L., Ansaldi F., Baldo V., Grassi R. (2020). Attitudes and Beliefs on Influenza Vaccination during the COVID-19 Pandemic: Results from a Representative Italian Survey. Vaccines.

[30] Ministry of Health, Government of Spain Flu 2020. Vaccines and Vaccination Program. Vaccination Coverage. https://www.mscbs.gob.es/profesionales/saludPublica/prevPromocion/vacunaciones/calendario-y-coberturas/coberturas/docs/Tabla13.pdf.

[31] Sebghati M., Khalil A. (2021). Uptake of vaccination in pregnancy. Best Pract. Res. Clin. Obstet. Gynaecol..

[32] Januszek S.M., Faryniak-Zuzak A., Barnaś E., Łoziński T., Góra T., Siwiec N., Szczerba P., Januszek R., Kluz T. (2021). The Approach of Pregnant Women to Vaccination Based on a COVID-19 Systematic Review. Medicina.

[33] To K.W., Lai A., Lee K.C.K., Koh D., Lee S.S. (2016). Increasing the coverage of influenza vaccination in healthcare workers: Review of challenges and solutions. J. Hosp. Infect..

[34] Smith S., Sim J., Halcomb E. (2016). Nurses’ knowledge, attitudes and practices regarding influenza vaccination: An integrative review. J. Clin. Nurs..

[35] Vilca L.M., Martínez C., Burballa M., Campins M. (2018). Maternal Care Providers’ Barriers Regarding Influenza and Pertussis Vaccination During Pregnancy in Catalonia, Spain. Matern. Child Health J..

[36] Mohammed H., Clarke M., Koehler A., Watson M., Marshall H. (2018). Factors associated with uptake of influenza and pertussis vaccines among pregnant women in South Australia. Berbers, G.A., ed. PLoS ONE.

[37] Lutz C.S., Carr W., Cohn A., Rodriguez L. (2018). Understanding barriers and predictors of maternal immunization: Identifying gaps through an exploratory literature review. Vaccine.

[38] Laenen J., Roelants M., Devlieger R., Vandermeulen C. (2015). Influenza and pertussis vaccination coverage in pregnant women. Vaccine.

[39] Dudley M.Z., Limaye R.J., Salmon D.A., Omer S.B., O’Leary S.T., Ellingson M.K., Spina C.I., Brewer S.E., Bednarczyk R.A., Malik F. (2021). Racial/Ethnic Disparities in Maternal Vaccine Knowledge, Attitudes, and Intentions. Public Health Rep..

[40] Okoli G.N., Reddy V.K., Al-Yousif Y., Neilson C.J., Mahmud S.M., Abou-Setta A.M. (2021). Sociodemographic and health-related determinants of seasonal influenza vaccination in pregnancy: A systematic review and meta-analysis of the evidence since 2000. Acta Obstet. Gynecol. Scand..

[41] Vilca L.M., Verma A., Buckeridge D., Campins M. (2017). A population-based analysis of predictors of influenza vaccination uptake in pregnant women: The effect of gestational and calendar time. Prev. Med..

[42] van Bakel H.J.A., Maas A.J.B.M., Vreeswijk C.M.J.M., Vingerhoets A.J.J.M. (2013). Pictorial representation of attachment: Measuring the parent-fetus relationship in expectant mothers and fathers. BMC Pregnancy Childbirth.

[43] Eurostat Young and Older Mothers in the EU. https://ec.europa.eu/eurostat/en/web/products-eurostat-news/-/DDN-20190801-1.

[44] Mak D.B., Regan A.K., Vo D.T., Effler P.V. (2018). Antenatal influenza and pertussis vaccination in Western Australia: A cross-sectional survey of vaccine uptake and influencing factors. BMC Pregnancy Childbirth.

[45] Benusic M., Cheskin L.J. (2021). Obesity prevalence in large US cities: Association with socioeconomic indicators, race/ethnicity and physical activity. J. Public Health.

[46] Maertens K., Braeckman T., Top G., Van Damme P., Leuridan E. (2016). Maternal pertussis and influenza immunization coverage and attitude of health care workers towards these recommendations in Flanders, Belgium. Vaccine.

[47] UK Health Security Agency (2022). Pertussis Vaccination Programme for Pregnant Women Update: Vaccine Coverage in England, July to September 2021. Health Protection Report. https://assets.publishing.service.gov.uk/government/uploads/system/uploads/attachment_data/file/1056185/FINAL-HPR0322-PRTSSS-vc-Q2_21022022.pdf.

[48] Maisa A., Milligan S., Quinn A., Boulter D., Johnston J., Treanor C., Bradley D.T. (2018). Vaccination against pertussis and influenza in pregnancy: A qualitative study of barriers and facilitators. Public Health.

[49] Sørensen K., Pelikan J.M., Röthlin F., Ganahl K., Slonska Z., Doyle G., Fullam J., Kondilis B., Agrafiotis D., Uiters E. (2015). Health literacy in Europe: Comparative results of the European health literacy survey (HLS-EU). Eur. J. Public Health.

[50] Phommachanh S., Essink D.R., Wright P.E., Broerse J.E.W., Mayxay M. (2021). Maternal health literacy on mother and child health care: A community cluster survey in two southern provinces in Laos. Fischer, F., Ed. PLoS ONE.

[51] Wilcox C.R., Bottrell K., Paterson P., Schulz W.S., Vandrevala T., Larson H.J., E Jones C. (2018). Influenza and pertussis vaccination in pregnancy: Portrayal in online media articles and perceptions of pregnant women and healthcare professionals. Vaccine.

[52] Hernández-García I., Giménez-Júlvez T. (2021). Youtube as a source of influenza vaccine information in spanish. Int. J. Environ. Res. Public Health.

[53] Yang D.X., Khera R., Miccio J.A., Jairam V., Chang E., Yu J.B., Park H.S., Krumholz H.M., Aneja S. (2021). Prevalence of Missing Data in the National Cancer Database and Association with Overall Survival. JAMA Netw. Open.

[54] Olivares B., Grimshaw-Aagaard S.L.S. (2019). Essential task or meaningless burden? Nurses’ perceptions of the value of documentation. Nord. J. Nurs. Res..

